# Proteolysis of the endothelial cell protein C receptor by neutrophil proteinase 3

**DOI:** 10.1111/j.1538-7836.2007.02480.x

**Published:** 2007-05-01

**Authors:** A VILLEGAS-MENDEZ, R MONTES, L R AMBROSE, A N WARRENS, M LAFFAN, D A LANE

**Affiliations:** * Department of Haematology, Imperial College London London, UK; ‡ Department of Immunology, Imperial College London London, UK; † Laboratory of Thrombosis and Haemostasis, Haematology Department and Division of Cardiovascular Sciences, Centre for Applied Medical Research, Clinica Universitaria/School of Medicine, University of Navarra Pamplona, Spain

**Keywords:** endothelial cells, EPCR, inflammation, neutrophils, PR3

## Abstract

**Background:**

The endothelial cell protein C receptor (EPCR) presents protein C to the thrombin:thrombomodulin complex on the endothelium of large vessels, and enhances the generation of activated protein C (APC) and activation of protease-activated receptor-1. A previous report has demonstrated binding of soluble (s) EPCR to activated neutrophils via surface proteinase 3 (PR3).

**Methods:**

We now report further characterization of this interaction. Activated neutrophils and purified PR3 both decrease endothelial cell (EC) surface EPCR, suggestive of its proteolysis.

**Results:**

When added to purified recombinant sEPCR, PR3 produced multiple cleavages, with early products including 20 kDa N-terminal and C-terminal (after Lys^176^) fragments. The binding of active site blocked PR3 to sEPCR was studied by surface plasmon resonance. Estimates of the *K*_D_ of 18.5–102 nm were obtained with heterogeneous binding, suggestive of more than a single interaction site.

**Conclusions:**

This work demonstrates PR3 binding to and proteolysis of EPCR and suggests a mechanism by which anticoagulant and cell protective pathways can be down-regulated during inflammation.

## Introduction

The protein C anticoagulant pathway is one of the major coagulation regulatory mechanisms and modulates thrombin generation by degrading factors (F) Va and VIIIa [1]. On binding thrombomodulin on the endothelial cell (EC) membrane, thrombin proteolytically activates protein C, whereas its specificity for fibrinogen is lost. Activation of protein C is greatly enhanced in the presence of the endothelial cell protein C receptor (EPCR) [2–4], a type I transmembrane glycoprotein related to the CD1/class I major histocompatibility complex superfamily [5, 6], of almost exclusive EC-specific distribution [7]. By binding EPCR with high affinity [8, 9], protein C is concentrated on the EC surface and the activation peptide orientated towards the active site of the thrombomodulin-thrombin complex, which increases severalfold the generation of activated protein C (APC) [3].

In addition to its anticoagulant role, EPCR has been shown to facilitate APC-mediated activation of protease-activated receptor-1 [10], which stimulates neuroprotective/anti-apoptotic signalling pathways [11]. This activity of EPCR may be critical in the beneficial clinical effects of APC in the treatment of severe sepsis, as a sublethal dose of *Escherichia coli* administered to baboons became lethal when animals were simultaneously administered an anti-EPCR monoclonal antibody (mAb) that blocks the binding of protein C to EPCR [12].

A soluble form of EPCR (sEPCR) is found in plasma [13], probably arising from proteolytic cleavage by a metalloproteinase [14]. sEPCR can bind to activated neutrophils via proteinase 3 (PR3) [15], a process partially dependent on the β-2 integrin Mac-1. PR3 is an elastinolytic neutral serine protease [16] stored in neutrophil granules which, following cell activation, is presented on the cell surface. PR3 is best known as the primary antigen of the antineutrophil cytoplasmic antibodies in the autoimmune vasculitis Wegener’s granulomatosis (WG). Elevated levels of sEPCR have been found in ∼24% patients with active WG [17], which could potentially favor interaction with PR3 and has been postulated to modulate the inflammatory response [15]. The interaction between EC-anchored EPCR and neutrophils has not been studied to date. If membrane EPCR is also able to bind to activated neutrophils, this would have interesting implications for the pathogenesis of inflammatory reactions because EPCR expression varies between different vascular beds. We report here further characterization of the previously reported interaction between sEPCR and PR3 and demonstrate that sEPCR is rapidly proteolyzed by PR3. Furthermore, we provide evidence for a similar effect on EC-bound EPCR by activated neutrophils. Our data strongly suggest a mechanism for down-regulation of anticoagulation during inflammation.

## Methods

### Expression and purification of soluble EPCR

Recombinant soluble (rs)EPCR incorporating a His tag was expressed in *Pichia pastoris* strain X-33 using the EasySelect Pichia expression kit (Invitrogen, Paisley, UK) broadly as described [18]. rsEPCR from yeast supernatant was purified first by affinity chromatography in a HiTrap™ Chelating HP column, followed by anion exchange purification on a HiTrap™ Q HP, mainly as previously described [13]. Purity was confirmed by sodium dodecylsulfate polyacrylamide gel electrophoresis (SDS–PAGE) followed by Simply Blue™ Safestain (Invitrogen) staining and the presence of rsEPCR by Western blot analysis with the anti-EPCR RCR-2 mAb (kindly provided by K. Fukudome, Saga Medical School, Japan).

### Neutrophil isolation

Human neutrophils were isolated from the citrated peripheral blood of healthy donors by density gradient centrifugation over Polymorphprep™ (Axis-shield PoC AS, Oslo, Norway). The upper (mononuclear) cell layer was discarded. The lower (neutrophil) layer was collected, washed, and contaminating erythrocytes lyzed by hypotonic lysis. Neutrophils were resuspended in RPMI 1640 medium (Invitrogen) supplemented with 100 U mL^−1^ penicillin, 100 μg mL^−1^ streptomycin, 2 mm l-glutamine (Invitrogen) and either 2% or 10% human male AB serum (Biowest, Ringmer, UK). Neutrophil purity (> 90%) was assessed by flow cytometry, and the lack of activation as a result of the isolation process was confirmed by by luminol-dependent chemiluminescence as described below.

### Flow cytometry

Cells were stained for flow cytometry using a standard procedure: 1 × 10^5^ cells were stained for 30 min at 4 °C with saturating concentrations of the relevant primary or secondary mAb (predetermined by titration, data not shown) and appropriate isotype-matched control mAbs of irrelevant antigenic specificity. After the final wash, cells were acquired and analyzed on a FACSCalibur (BD Biosciences, San Jose, CA, USA) using cellquest software (BD Biosciences). Specifically, neutrophil purity or presence was assessed using a combination of forward and side scatter characteristics, and labelling with mouse antihuman CD16 PE or CD16 FITC (Caltag, Burlingame, CA, USA). EPCR expression on EA.hy926 cells, an endothelium-derived cell line expressing EPCR [19] (a kind gift from Cora-Jean Edgell, University of North Carolina, Chapel Hill, NC, USA), was assessed by labelling with the rat antihuman EPCR mAb RCR-252 (Hycult Biotechnology, Uden, The Netherlands), or the isotype-matched rat IgG1 control mAb (BD Biosciences), and an antirat FITC-conjugated secondary mAb (BD Biosciences).

### Neutrophil activation

Neutrophil activation as a result of either the isolation process or incubation with 50 ng mL^−1^ phorbol-12-myristate 13-acetate (PMA; Sigma-Aldrich, Gillingham, UK) was measured by luminol-dependent chemiluminescence. 1 × 10^5^ neutrophils were plated in triplicate in a 96-well plate (Costar, Helena Biosciences, Sunderland, UK) as follows: (i) negative control, neutrophils alone; (ii) positive control, neutrophils, 5 × 10^−4^ m luminol (Sigma-Aldrich) and 50 ng mL^−1^ phorbol 12-myristate 13-acetate (PMA, Sigma); (iii) test sample, neutrophils and 5 × 10^−4^ m luminol. The plate was read by a chemiluminometer (Lucy1; Anthos Labtech, Austria), which measured emission of light (arbitrary light units) per unit time at 180-s intervals for 2 h. Light emission was plotted on a light vs. time graph. The data were analyzed using stingray software (Dazdaq, Brighton, UK).

### Co-culture of EA.hy926 with activated neutrophils

EA.hy926 cells were grown to confluence (∼1 × 10^6^ cells well^−1^) on 9.6 cm^2^ diameter six-well plates and incubated with or without freshly isolated, PMA-activated neutrophils (3 × 10^6^ well^−1^, previously washed to exclude EPCR cleavage by PMA) for 0, 1, 2 and 24 h. After each time-point, EC were washed of neutrophils and harvested by incubation with a non-enzymatic buffer (10 mm EDTA in phosphate buffered saline, pH 7.4) for 30 min at 37 °C. The surface expression of EPCR on EA.hy926 cells at each time point after either co-culture with activated neutrophils or culture alone was measured by flow cytometry using the anti-EPCR mAb RCR-252, as described above. Where appropriate, the presence of residual neutrophils was assessed by staining the EC for CD16.

### Incubation of EA.hy926 with purified PR3

EA.hy926 cells were harvested from confluent cultures as described above. 1 × 10^6^ cells were subsequently incubated with 100 or 250 nm of PR3 (purity > 95%, Elastin Products Co., Inc., Owensville, MO, USA) in human endothelial-serum free medium (Invitrogen) supplemented with 20 mm HEPES (Invitrogen) for 6 h at 37 °C. The surface expression of EPCR on EA.hy926 cells after incubation with PR3 was measured by flow cytometry using the anti-EPCR mAb RCR-252, as described above. Dead cells were excluded from the analysis by staining with propidium iodide immediately prior to acquisition on the FACSCalibur (BD Biosciences).

### Proteolysis of rsEPCR by PR3

PR3, 1 μm, was incubated with 10 μm rsEPCR at 37 °C, either in HBS-P buffer or in pooled human plasma (Technoclone, Surrey, UK). Aliquots were taken at 0, 5, 15, 30, 60 and 180 min and the fragmentation of rsEPCR was analyzed by SDS–PAGE in 16.5% Tris–Tricine gels (Bio-Rad, Hertfordshire, UK) under reducing conditions, followed by Simply Blue™ Safestain staining and Western blotting using anti-His (C-term) mAb (Invitrogen).

### HPLC analysis of rsEPCR cleavage by PR3

rsEPCR (5 μm) was incubated with 0.5 μm PR3 in reaction buffer (10 mm HEPES, 150 mm NaCl, pH 7.4). Reactions were stopped after 0, 15, 30, 60, 120, 240 and 390 min by addition of hundredfold excess of phenyl methyl sulfonyl fluoride (PMSF) relative to PR3. Samples were separated by reversed phase chromatography in an ÄKTA™ basic purifier system (Amersham Biosciences, Little Chalfont, UK) using a 5% to 75% linear gradient of acetonitrile in a 150 mm length Biobasic C4 column (Thermo Electron Corporation, Basingstoke, UK).

### Proteomic analysis of rsEPCR cleavage by PR3

rsEPCR (250 pmol) was incubated with 25 pmol PR3 in 5 mm ammonium bicarbonate, pH 7.8 for 15 min. Samples were separated by SDS–PAGE and stained with Simply Blue™ Safestain. Gel bands were excised and transferred to Packard robot, where they were automatically reduced and carbamidomethylated, following treatment with trypsin, as described elsewhere [20, 21]. The trypsinized samples were transferred to glass vials for CapLC-MSMS analysis using an LC packings PepMap column (10 cm × 75 μm, 70 Å). Samples were analyzed with MALDI-ToF MS on a MALDI instrument (Micromass, Altrincham, Cheshire, UK) using recrystallized alpha-cyano-4-hydroxycinnamic acid as matrix. MSMS data were obtained on a Micromass QtoF II instrument, either directly using a nanospray needle or by capillary LC-MSMS. MS and MSMS data were converted to Micromass ‘pkl’ files for analysis by the MASCOT search engine (http://www.matrixscience.com) using the non-identical MSDB protein data base. Individual MSMS spectra were also analyzed manually. Masses of candidate protein fragments were matched with the EPCR sequence using micromass biolynx software.

### Binding affinity of PR3 for rsEPCR

All binding experiments were investigated by SPR using a dual flowcell BIAcore® X Biosensor system (Biacore, AB, Uppsala, Sweden). For direct immobilization analysis, 35 μL of 60 μg mL^−1^ sEPCR solution in 10 mm sodium acetate pH 5.0 was immobilized on one flow cell of a CM-5 sensor chip, to give a response in resonance units (RU) of 2350. PR3 (1 μm) was diluted in 50 mm HEPES pH 7.4, 150 mm NaCl (HBS-P, Biacore) and injected over the sEPCR surface. All subsequent SPR analyses were performed using a mAb approach as described elsewhere [22]. Immobilization was performed by injection of 30 μL of 10 μg mL^−1^ anti-His C-terminal mAb (Invitrogen) solution in 10 mm sodium acetate pH 5.0 over both flow cells of a CM-5 sensor chip. A response of between 8000 and 10 000 RU was established. Subsequently, 50 μL of rsEPCR diluted in HBS-P was injected and equilibrated over flow cell 2 only, until the response level was between 850 and 1000 RU. The flow cell without rsEPCR bound was used as a reference cell. PR3 (0–300 nm) in HBS-P were sequentially injected over both flow cells at a flow rate of 20 μL min^−1^ for 200 s. PR3 was allowed to completely dissociate from the rsEPCR surface before the next PR3 injection. In some experiments 1 mm PMSF was included in the PR3 dilutions and in the running buffer to inhibit PR3 activity. Any influence of mass transport effects was excluded from the sensograms based on the equal binding curves obtained when injecting 300 nm PR3 at different flow rates.

Data analysis was performed using the biaevaluation software 3.0 (Biacore). All data sets were corrected for non-specific binding (reference cell subtraction, flowcell 2–flowcell 1) and for refractive index changes (zero subtraction). Kinetics of PR3 binding to rsEPCR were fitted globally to the heterogeneous ligand binding model. A second estimation of the *K*_D_ was obtained based on the RU values at binding equilibrium (*R*_eq_) fitted to the steady state binding model.

## Results

### Activated neutrophils decrease EPCR on EC

**Fig. 1:**
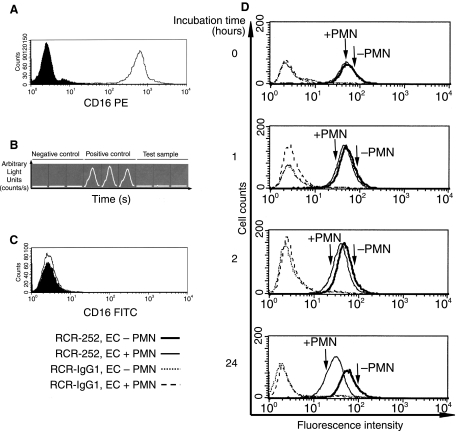
Loss of endothelial cell protein C receptor (EPCR) expression on endothelial cell (EC) after incubation with activated neutrophils. (A) Purity of freshly isolated neutrophils. Neutrophil purity, as detected by flow cytometry using a PE-conjugated anti-CD16 monoclonal antibody (mAb) (open histogram) and an isotype-matched control mAb, was routinely greater than 90%. (B) Activation status of freshly isolated neutrophils. Freshly isolated neutrophils, tested in triplicate, were unactivated by the isolation procedure (test sample), but capable of responding to the stimulus PMA (positive control), as measured by luminol-dependent chemiluminescence for 7200 s. The cells were activated with 50 ng mL^−1^ PMA for 30 min prior to incubation with EA.hy926 cells. (C) Efficient removal of activated neutrophils after co-culture with EA.hy926 cells. The lack of contaminating residual neutrophils within the EC population after co-culture was confirmed by flow cytometry for CD16 FITC (open histogram) and an isotype-matched control mAb (shaded histogram). The decreased EPCR expression observed in Fig. 1D therefore reflects a *bona fide* decrease in the EC EPCR expression and is not because of a population of adherent (EPCR-negative) neutrophils. (D) Surface expression of EPCR after incubation with activated neutrophils. EA.hy926 cells were incubated with or without activated neutrophils for 0, 1, 2 and 24 h. The EPCR expression level on EC after neutrophil treatment (thin solid line) or medium only treatment (thick solid line) was measured by flow cytometry with the mAb RCR-252 or an isotype-matched rat IgG_1_ control (discontinuous lines). The EC treated with activated neutrophils showed a time-dependent reduction of EPCR expression, observed as a decrease in the mean fluorescence intensity from 49.7 to 28.8 between 0 and 24 h.

To investigate whether activated neutrophils are able to interact with EC surface EPCR, freshly isolated neutrophils (> 90% purity, Fig. 1A), unactivated by the isolation procedure but fully responsive to activation by PMA (Fig. 1B) were activated with PMA prior to incubation with EA.hy926 cells. Control EA.hy926 cells were cultured under identical conditions but without neutrophils. In both cases, surface EPCR expression was measured over time by flow cytometry (Fig. 1D) with the mAb RCR-252. The EC treated with activated neutrophils showed a time-dependent reduction of EPCR expression, observed as a decrease in the mean fluorescence intensity (MFI) from 49.7 to 28.8, between 0 and 24 h (Fig. 1D, solid thin lines). Similar results were obtained when EPCR expression was detected by another antihuman EPCR mAb, RCR-2, recognizing a separate epitope [22] (data not shown). The lack of contaminating residual neutrophils adherent to the EC was confirmed by flow cytometry for CD16 FITC (Fig. 1C). When EC were incubated with non-activated neutrophils, no decrease in surface EPCR expression was observed (data not shown). These results suggest that EPCR on the EC is cleaved by a protease present on the surface of activated neutrophils, although other mechanisms of down-regulation are not excluded. Of the protease repertoire expressed on activated neutrophils, PR3 has previously been shown to interact with sEPCR [15] and is therefore a candidate cause.

### PR3 degrades membrane EPCR

**Fig. 2:**
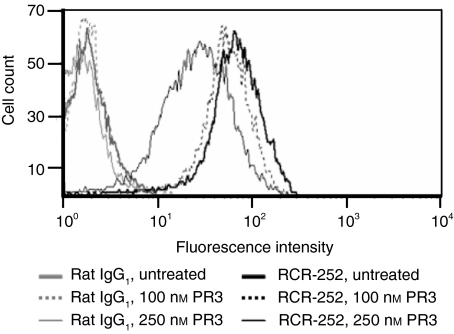
Degradation of membrane endothelial cell protein C receptor (EPCR) by PR3. EA.hy926 cells were incubated for 6 h with 0 (untreated), 100 and 250 nm PR3. Surface EPCR expression was detected with RCR-252 by flow cytometry, using an isotype-matched rat IgG_1_ mAb as a control. A representative experiment of four independent repeats is shown.

Purified PR3 from human leukocytes was incubated with EA.hy926 cells. Cells were incubated with increasing concentrations of PR3 for 6 h and EPCR detected with RCR-252. EPCR expression decreased only in cells that had been incubated with PR3 (Fig. 2) in a time- and dose-dependent manner. After 6 h, the MFI decreased from 61.0 to 50.6 (100 nm PR3, broken line RCR-252) and 24.5 (250 nm PR3, thin intact line RCR-252). These data suggest that the loss of EPCR on EC after neutrophil treatment can be ascribed in part to the action of PR3.

### Proteolysis of rsEPCR by PR3

**Fig. 3:**
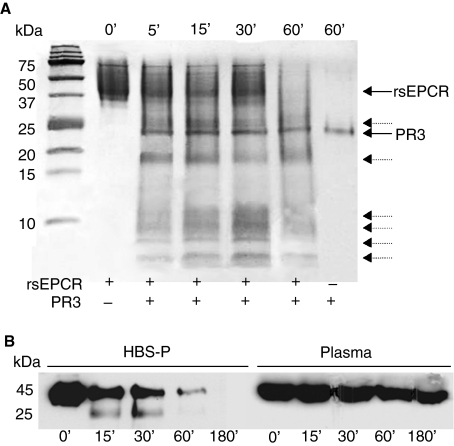
Recombinant soluble endothelial cell protein C receptor (rsEPCR) proteolysis by PR3. (A) sodium dodecylsulfate polyacrylamide gel electrophoresis (SDS–PAGE) analysis. PR3 and rsEPCR (1:10 molar ratio) were incubated in HBS-P at 37 °C for 0, 5, 15, 30 and 60 min and protein bands resolved by SDS–PAGE under reducing conditions in 16.5% Tris-Tricine gels. Solid arrows indicate the migration of PR3 and intact rsEPCR. Dotted arrows indicate degradation products of rsEPCR. (B) Western blot analysis. rsEPCR was incubated with PR3, as described above, in HBS-P buffer or with pooled human plasma for 0, 15, 30, 60 and 180 min. Samples were subsequently blotted and analyzed with anti-His (C-term) mAb.

Cleavage of rsEPCR was followed directly over time by SDS–PAGE analysis. As shown in Fig. 3A, PR3 rapidly cleaved rsEPCR at 1:10 molar ratio. Degradation bands of different Mr (ranging from ∼25 to < 10 kDa) could be seen after 5 min incubation with PR3, which is not observed in the control sample of rsEPCR (Fig. 3A, time-point ‘0’, note that the heterogeneity is a result of different glycosylation on the EPCR molecule). This is indicative of cleavage taking place at several sites in rsEPCR (Fig. 3A). After 60 min of incubation with PR3 no intact rsEPCR remained. Accordingly, when a mAb against the His C-terminus was used in the Western blot analysis, a progressive degradation of rsEPCR could be observed. A novel band of 25 kDa was temporarily detected, again suggesting proteolytic cleavage at several sites (Fig. 3B). When the experiment was performed in the presence of pooled human plasma, only a very weak degradation of rsEPCR could be seen, compatible with the presence of PR3 inhibitors in plasma, such as α_1_-antitrypsin [23] (Fig. 3B).

**Fig. 4:**
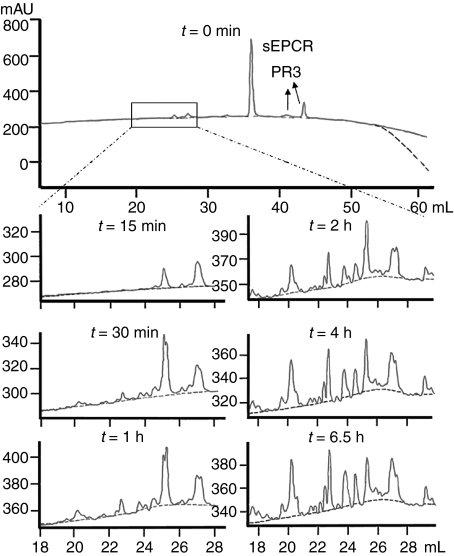
High pressure liquid chromatography (HPLC) time-course analysis of recombinant soluble endothelial cell protein C receptor (rsEPCR) proteolysis by PR3. rsEPCR (250 pmol) were incubated with PR3 (25 pmol) and a time-course analysis of cleavage was monitored by HPLC in a Biobasic C4 column, using a 5–75% acetonitrile gradient. The sEPCR peptides released upon PR3 cleavage over time (15 min to 6.5 h) are shown in the zoomed chromatograms. O.D._205 nm_ is expressed in mAU units; retention volumes are shown in mL.

Cleavage of rsEPCR was also investigated by high pressure liquid chromatography (HPLC) analysis over time after direct incubation of PR3 with rsEPCR (molar ratio 1:10). At the start of the incubation (Fig. 4, top panel), rsEPCR eluted at 55% of the acetonitrile gradient as a single peak and PR3 at 60 and 75% acetonitrile as two peaks. Proteolysis can be observed as an elution of multiple peaks increasing over time (Fig. 4, zoomed area), which coincided with the disappearance of the undigested rsEPCR peak (not shown). These data are indicative of PR3 cleavage at different sites with the subsequent release of multiple peptides from rsEPCR.

### Proteomic analysis of rsEPCR cleavage

**Fig. 5:**
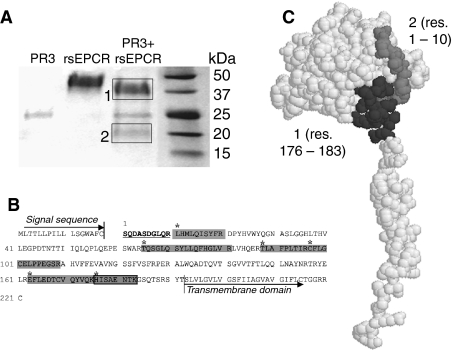
Proteomic analysis of recombinant soluble endothelial cell protein C receptor (rsEPCR) cleavage. (A) rsEPCR (250 pmol) were incubated with PR3 (25 pmol) for 15 min and electrophoresed on a 16% Tris–Tricine gel. Protein bands were trypsinized and analyzed by MALDI-ToF. Lane 1, PR3; lane 2, undigested rsEPCR; lane 3, rsEPCR + PR3, resulting in bands of ∼40 kDa (fragment 1) and 20 kDa (fragment 2); lane 4, protein markers. (B) Protein identification by MASCOT data base search using the trypsinized bands from (A). Matching peptides (first residue indicated with asterisk) corresponding to EPCR are highlighted in grey. The sequence in bold underlined was identified in fragment 2 by manual searching ignoring the signal peptide sequence, whereas the sequence in the box was only identified in undigested rsEPCR. (C) Molecular model of full-length EPCR generated by Rasmol 2.6 using the coordinates provided by Villoutreix *et al.* [41]. Areas of predicted cleavage are highlighted as regions 1 (residues HISAENTK, 176–183 of EPCR) and 2 (residues SQDASDGLQR, 1–10 of EPCR).

rsEPCR was digested with PR3 and the samples run on a non-reducing SDS–PAGE, followed by Simply Blue™ Safestain staining. Undigested EPCR was detected as a ∼45 kDa band (Fig. 5A, lane 2), and the digestion products as a ∼40 kDa band (Fig. 5A, lane 3, band 1) and a ∼20 kDa band (Fig. 5A, lane 3, band 2). Given the Mr of rsEPCR (45 kDa), another band of ∼25 kDa was expected after cleavage with PR3. It is possible that this band runs simultaneously with the diffuse band of ∼20 kDa, or that it gets further proteolyzed into smaller components and hence is not detected in the gel. All bands, including PR3 (Fig. 5A, lane 1), undigested rsEPCR and the two digestion products detected were excised, reduced/carbamidomethylated and trypsinized under conditions appropriate for proteomics analysis. The extracted products were analyzed by capillary LC-MSMS on a QtoF mass spectrometer in the survey mode giving both MS and MSMS data. The band from lane 1 was confirmed to be PR3, while the band from lane 2 matched the sEPCR sequence, with six peptides identified providing good coverage of its sequence (Fig. 5B). Peptides containing predicted post-translational N-linked modifications could not be identified. Fragment 1 of rsEPCR (Fig. 5A, lane 3, band 1) gave similar peptide coverage to sEPCR using the MASCOT search, except for sequence HISAENTK (176–183 of EPCR, see Fig. 5B, sequence in box and Fig. 5C, region 1), which was not detected, suggesting cleavage took place after position 176 of sEPCR (C-terminus cleavage). N-terminal peptide search (excluding predicted matching against the sequence containing the signal peptide) on fragment 2 of rsEPCR (Fig.5A, lane 3, band 2) identified the sequence SQDASDGLQR, corresponding to residues 1–10 of sEPCR (Fig 5B, bold underlined sequence and Fig. 5C, region 2). This finding implies that the 20 kDa rsEPCR cleavage product arises from internal cleavage of sEPCR and contains the original N-terminus of the protein. This methodology provided evidence of early proteolysis in adjacent residues of the C-terminus of sEPCR and an internal region (Fig. 5C).

### Affinity of PR3 for rsEPCR

**Fig. 6:**
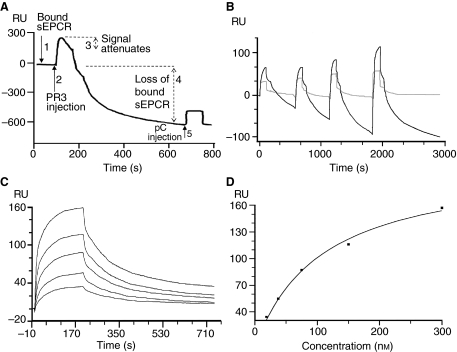
SPR analysis of binding between PR3 and rsEPCR (endothelial cell protein C receptor). (A) Loss of protein C binding after rsEPCR exposure to PR3. (i) Immobilization of rsEPCR in one flow cell of a CM-5 sensor chip (2300 RU), followed by (ii) injection of 1 μm PR3. Proteolysis of rsEPCR can be appreciated at the end of the association phase (iii) and during the dissociation phase (iv). (v) Injection of 300 nm protein C after sEPCR proteolysis. (B) Individual flow cell display of PR3 binding to rsEPCR. The anti-His mAb was immobilized on a CM-5 sensor chip (∼10 000 RU in both flow cells) to allow the capture of rsEPCR. rsEPCR was injected across flow cell 2 (∼1000 RU), followed by injection of active PR3 (concentration range from 0 nm to 200 nm) across both flow cells for 100 s at 20 μL min^−1^ flow rate. Note that a decrease in the baseline level is only observed in flow cell 2 (black sensogram), where rsEPCR is immobilized. (C) Binding of phenyl methyl sulfonyl fluoride (PMSF) inactivated PR3 to anti-His captured rsEPCR. The experiment was performed as described in (B), except for the addition of 1 mm PMSF to inactivate PR3. Increasing concentrations of PMSF inactivated PR3 (range between 0 nm–300 nm) were injected on the sensor chip surface for 200 s contact time. (D) Binding of PR3 to rsEPCR as assessed by the steady state model. The *R*_eq_ (180 s of the association phase) from each binding curve represented in (C) were plotted against the corresponding concentration of PR3 to generate a saturation binding curve. The *K*_D_ was calculated using the steady state model from the biaevaluation software 3.0.

Direct immobilization of ligand molecules was initially performed to obtain a first indication of the binding between rsEPCR and PR3 by SPR. sEPCR was immobilized onto a sensor chip (Fig. 6A, arrow 1) and 1 μm of active PR3 injected over the chip (Fig. 6A, arrow 2). Using this high concentration of PR3, it was apparent the aberrant dissociation during the association phase (Fig. 6A, arrow 3), which fell well below (∼700 RU) the baseline reading (Fig. 6A, arrow 4). Interestingly, the injection of protein C to the rsEPCR exposed to PR3 failed to bind specifically (Fig. 6A, arrow 5). To avoid the heterogeneity of direct coupling and the previously reported loss of sEPCR activity because of direct binding to artificial surfaces [24], an antibody approach was adopted. The anti-His C-terminal mAb was first immobilized onto the surface of a CM5 sensor chip and used to capture rsEPCR expressed in *P. pastoris* with a 6-histidine tag at its C-terminus. Non-specific binding of PR3 to the control flow cell can be seen with each PR3 injection (Fig 6B, grey sensorgram). In the test cell, PR3 could be seen binding to rsEPCR in a concentration-dependent manner and displacing it from the anti-His mAb, the latter observed as a drop in the baseline level of rsEPCR with the sequential injections of PR3 (Fig. 6B, black sensogram). This loss of rsEPCR from the mAb surface and that observed in the direct immobilization method (Fig. 6A, arrow 4) were attributed to proteolysis of rsEPCR by PR3.

To prevent loss of rsEPCR from the sensor chip surface, experiments were also performed with PMSF inactivated PR3 (Fig. 6C). Use of the 1:1 Langmuir model resulted in poor fitting, suggesting that the interaction of sEPCR and PR3 does not follow a simple 1:1 binding. In order to obtain a first estimate of the binding affinity, the *R*_eq_ from each concentration of PR3 injected over the surface containing rsEPCR, were used on the steady state fit model (Fig. 6D). Using this model, the *K*_D_ for the interaction of PR3 and rsEPCR was found to be 102 ± 12.6 nm (*n* = 3). In order to obtain detailed information on the association and dissociation phase of the interaction, different fitting models were considered. Optimal fitting was found using the heterogeneous ligand model (Fig. 6C). This model assumes the ligand (rsEPCR) molecule is heterogeneous with regard to analyte (PR3) binding and approximates the different range of interactions using two sets of rate constants. Analysis yielded a *k*_a1_ of (3.90 ± 0.27) ×10^5^ m^−1^ s^−1^, a *k*_d1_ of (1.57 ± 0.23) × 10^−2^ s^−1^, resulting in a *K*_D1_ of 40.2 ± 4.2 nm and a *k*_a2_ of (8.78 ± 3.59) ×10^4^ m^−1^ s^−1^, a *k*_d2_ of (1.47 ± 0.08) × 10^−3^ s^−1^, resulting in a *K*_D2_ of 18.5 ± 6.4 nm (*n* = 3). Alternative models (heterogeneous analyte or conformational change) were excluded as valid, given the equal dissociation curves obtained after injection of a high concentration of PR3 (2 μm) at different contact times (2.5 and 5 min), which suggests the presence of only one population of analyte (data not shown).

## Discussion

In addition to its role as an autoantigen in WG, PR3 is known to have a diverse range of activities in the regulation of inflammation, including degradation of extracellular matrix proteins [23], potentiation of platelet activation [25] and processing proinflammatory cytokines and receptors to mature forms (TNF-α, IL-1β, TGF-β, IL-18 [26–28]). PR3 has previously been shown to bind sEPCR on the surface of activated neutrophils [15]. sEPCR is generated from EC-bound EPCR by metalloproteolytic release, a highly regulated process that is sensitive to both coagulation factors (thrombin) and inflammatory mediators (IL-1β) [14]. The significance of the sEPCR-PR3 interaction, and its implications for the function of sEPCR and of its EC-anchored counterpart have not been elucidated to date. In this study, we demonstrate that the binding between rsEPCR and PR3 is a high affinity process of a complex nature, which results in the proteolytic cleavage of rsEPCR. We also report for the first time, evidence for a similar interaction between PR3 on activated neutrophils and EC-anchored EPCR that results in proteolysis of the intact membrane receptor.

We have shown previously that rsEPCR binding to protein C is a process characterized by fast association and dissociation binding rates, resulting in an overall *K*_D_ of 74.8 nm [22].The binding of rsEPCR to PR3 is also found to be of high affinity (Fig. 6C), with overall *K*_Ds_ of 40.2 and 18.5 nm. These latter binding interactions may be considered of doubtful significance because the plasma concentration of sEPCR is ∼2.5 nm and is elevated only 2- to 5-fold in disease [13, 29]. The demonstration here that incubation of EA.hy926 cells with PMA activated neutrophils resulted in loss of surface EPCR expression is therefore likely to be of greater importance. We presume that binding of activated neutrophils to EC via a number of established mechanisms produces effective high local concentrations of EPCR and PR3 favoring interaction and proteolysis of membrane EPCR. The β-2 integrin Mac-1, known to interact with PR3 on the neutrophil membrane [30], was also found to be partially involved in the interaction with sEPCR [15]. Thus, an increase of Mac-1 levels (as reported in the case of active WG patients [31]) would favor local accumulation of primed neutrophils on the endothelium at sites of vascular injury. Although we have not formally investigated the role of Mac-1 in the binding to EC EPCR, it is plausible that this integrin further localizes the EPCR interaction with PR3 on the activated neutrophil surface. However, the potential for the EPCR interaction with PR3 will vary significantly between different vascular beds because of the variable expression of EPCR within the vasculature.

PR3 is expressed at detectable levels in the membrane of resting neutrophils [32, 33] and is rapidly up-regulated by proinflammatory mediators, leading to expression of PR3 on the neutrophil surface. This bound PR3 is resistant to inhibition by plasma proteinase inhibitors, in marked contrast to soluble PR3 [33]. Our finding of plasma inhibition of PR3 mediated sEPCR proteolysis (Fig. 3B) is consistent with this and is another indicator that the important interaction between these two molecules is likely to involve their cell surface bound forms.

The extensive proteolysis of EPCR by PR3 that we have demonstrated *in vitro* strongly suggests that a role of PR3 is to abolish EPCR activity. In support of this hypothesis, SPR experiments indicate that sEPCR does not bind protein C after proteolysis by PR3 (Fig. 6A). Moreover, the cleavage appeared to be specific for EPCR, as PR3 failed to cleave immobilized anti-His mAb in the control flow cell (see Fig. 6B, grey sensogram) and other mAbs or control proteins (thrombin) used during the SPR optimization process (data not shown). Such action of PR3 from activated neutrophils on EC-anchored EPCR is likely to be of pathophysiological importance. In addition to its role in regulation of coagulation activity, APC inhibits leukocyte adhesion to vascular EC and proinflammatory cytokine release from monocytes [34], reduces accumulation of neutrophils in rat lungs [35] and protects baboons from *E. coli*-induced sepsis. At the cell molecular level, APC modulates patterns of gene expression of the anti-inflammatory, cell survival and apoptosis pathways in EC [36]. In addition, other mechanisms in combination with proteolysis could account for the decreased EPCR levels. EC can internalize PR3 and activate proapoptotic signaling pathways [37–39] in response to vascular injury [40]. It can be hypothesized that the high affinity of EPCR for PR3 on the EC surface might contribute to localizing activated neutrophils in the damaged area and trigger proteolysis of EPCR, facilitating internalization of PR3. Depletion of EPCR on the EC surface during acute inflammation might therefore accentuate the inflammatory process.

The finding of cleavage at the C-terminus of sEPCR between Lys^175^ and His^176^ (Fig. 5B), suggests that at least one of the initial binding sites of PR3 on sEPCR might be located in its C-terminal region. This is compatible with protein C or APC not affecting significantly the sEPCR-PR3 interaction [15]. The multiple cleavage sites demonstrated by SDS–PAGE (Fig. 3A), HPLC (Fig. 4) and proteomic analysis (Fig. 5A,B) suggest a complex interaction between sEPCR and PR3, observed also by SPR (Fig. 6C), with PR3 binding on different regions of sEPCR. This is therefore an unlikely target for potential pharmacological inhibition.

We have confirmed a high affinity interaction between the neutrophil protease PR3 and the EPCR, which results in the proteolytic degradation of the receptor. Further experiments have shown that *in vivo* this is likely to be mediated by cellular interaction involving the surface bound forms of the molecules. Degradation of EPCR with consequent loss of APC generation is likely to contribute to the already known proinflammatory roles of PR3.
